# The mediating role of optimism between reading habits and meaningful living

**DOI:** 10.3389/fpsyg.2025.1573682

**Published:** 2025-04-02

**Authors:** Ahmet Zeki Guven, Emrullah Banaz, Uğur Özbilen, Tuğrul Gökmen Şahin

**Affiliations:** ^1^Department of Turkish Language Education, Faculty of Education, Akdeniz University, Antalya, Türkiye; ^2^Department of Education Programs and Teaching, Faculty of Education, Bayburt University, Bayburt, Türkiye; ^3^Independent Researcher, Kilis, Türkiye; ^4^Department of Turkish Language Education, Faculty of Education, İnönü University, Malatya, Türkiye

**Keywords:** meaningful living, optimism, positive psychology, reading habits, structural equation modeling

## Abstract

The present study sought to examine the relations between reading habits, perceived meaningful living, and optimism through structural equation modeling. To this end, we recruited undergraduate students enrolled in the Turkish language teaching program of a state university and collected the data using the Reading Attitude and Habits Scale, the Meaningful Living Scale and the Optimism-Pessimism Questionnaire. More than half (58.4%) of students were females, 52% were aged 21–30 years, and 34.2% were second-year students. The data were analyzed using SPSS 24.0 and SPSS AMOS 20.0. The findings showed a significant relationship between reading habits and meaningful living, with optimism serving as a significant mediator. Model fit indices confirmed the validity of the SEM model (RMSEA = 0.061, CFI = 0.94, TLI = 0.96). We also found that optimism served as a significant mediator in the relation between reading habits and perceived meaningful living. The findings suggest encouraging undergraduate students’ reading habits and designing and offering counseling services to foster students’ optimism and perceived meaningful living. Moreover, further research with diverse demographic groups is needed to elucidate more on the subject.

## Introduction

The pursuit of meaning in life exerts a substantial influence on one’s life satisfaction and psychological wellbeing. In other words, the quest for meaningful living is a profound need that stems from the desire to find purpose and value in life, which is an integral facet of wellbeing. In this sense, meaningfulness is inextricably linked to one’s recognition of the purpose and value of their life (Steger et al., 2006). Therefore, it can be posited that the components dictating one’s perceived meaningful living may significantly affect their overall psychological state. In particular, personality traits, such as optimism and pessimism, are prominent psychological factors influencing how one makes sense of their life. It is noteworthy that optimistic individuals tend to perceive undesirable life events as more manageable in comparison to their pessimistic counterparts (Carver and Scheier, 2005).

A multitude of factors contribute to one’s perception of meaningful living, and reading habits may be considered the leading one among these factors. The practice of reading is consistently shown to facilitate a more expansive perception of life, cultivate empathy, and enhance self-understanding (Billington et al., 2017). It also plays an instrumental role in individuals acquiring knowledge, investing in personal growth, and adding meaning to their lives (Clark and Rumbold, 2006). Reading habits are regarded as a pivotal means of mental development, learning, and self-awareness. Hence, tracing the association between reading habits and perceived meaningful living has emerged as a significant research area, intending to enhance one’s psychological wellbeing and positive self-perceptions. A substantial body of research demonstrated that reading not only contributes to cognitive development but also facilitates a more emotionally and socially fulfilling life (Guthrie and Wigfield, 2000). Previous research showed that reading habits have a significant impact on academic achievement and subjective wellbeing (Krashen, 2004). Reading allows individuals to enhance their self-understanding and improve their ability to grasp their environment, thereby contributing to developing a stronger sense of meaning in their own lives (Nell, 1988). As meaningful living is particularly associated with one’s perception of their life as valuable, purposeful, and meaningful (Steger et al., 2006), engaging in regular reading may support a higher level of perceived meaningful living. Reading habits shape individuals’ perception of the world while also influencing their levels of optimism. Studies have shown that individuals with regular reading habits tend to have a broader perspective and develop a more positive outlook on life (Krashen, 2004). In particular, it is known that literary works and self-improvement books enhance psychological resilience and help individuals evaluate life events from a more positive perspective. In this context, the relationship between reading habits and optimism can be seen as a critical factor contributing to an individual’s perception of a meaningful life. Understanding how reading habits contribute to meaningful living can have important educational and psychological implications. Given that university students are at a crucial stage of identity formation and career development, fostering reading habits may enhance their ability to find purpose in life.

This study is based on Frankl’s (2021) logotherapy theory, which posits that meaning-making is a central aspect of psychological health. Additionally, Seligman’s (2002) positive psychology framework suggests that optimism plays a critical role in wellbeing, which aligns with our hypothesis that optimism mediates the relationship between reading habits and meaningful living. The pursuit of meaning in life exerts a substantial influence on one’s life satisfaction and psychological wellbeing. Meaningful living is closely linked to individuals’ recognition of purpose and value in life (Steger et al., 2006). The present study investigates the role of reading habits in fostering meaningful living among university students, focusing on optimism as a potential mediator. University students are in a critical period of self-discovery, and their reading habits can significantly shape their worldviews, coping strategies, and psychological resilience.

The principal objective of this study was to examine the relations between reading habits, perceived meaningful living, and optimism among prospective Turkish language teachers through structural equation modeling (SEM). SEM is often considered an effective approach for analyzing intricate relations and facilitates uncovering both direct and indirect effects between variables (Byrne, 2013). Prior studies have shown that reading habits contribute to cognitive and emotional development, fostering a more profound sense of meaning in life (Clark and Rumbold, 2006). Carver and Scheier (2005) found that frequent reading enhances empathy and social understanding. Additionally, optimism has been associated with greater resilience and well-being. This study builds upon these findings by examining how optimism mediates the relationship between reading habits and meaningful living. Overall, we sought the link between perceived meaningful living and reading habits and to uncover the mediating effect of optimism in this relation. Given that meaningful living is not only an individual experience but also a dynamic process tailored by cultural and social interactions, our findings are likely to bring substantial contributions to psychological wellbeing, education, culture, and personal development (Frankl, 2021). Therefore, we proposed the following hypotheses:

*H1:* Reading habits positively affect perceived meaningful living.

*H2:* Optimism positively affects perceived meaningful living.

*H3:* Reading habit positively affects perceived meaningful living through optimism.

## Methods

This cross-sectional correlational research examined the relations between reading habits, optimism, and perceived meaningful living through SEM. We deemed SEM appropriate for attaining our research objective, as it allows for testing both direct and indirect relations between variables of interest.

### Participants

This study employed a convenience sampling method due to accessibility constraints. We performed a preliminary power analysis using the G*Power 3.1.9.7 program (Faul et al., 2007) to determine the sample size to test our hypotheses. Accordingly, we calculated the minimum sample size necessary to detect a medium effect (d = 0.15; Cohen, 2013) and to attain 95% power at the significance of *α* = 0.05 to be 107. While this study is exploratory in nature, the potential implications of the findings warrant a higher power and significance level to minimize the risk of overlooking a potentially crucial effect. Therefore, we selected 201 undergraduate students enrolled in the Turkish language teaching program at Akdeniz University. We employed convenience sampling to ensure diversity in the demographic characteristics of participants. All participants provided written informed consent before the data collection procedure.

### Measures

#### Reading attitude and habits scale

Özdil and Demir (2020) designed the 19-item Reading Habits and Attitude Scale (RHAS) and confirmed its 2-factor structure (*χ^2^* = 401.62, df = 266, RMSEA = 0.061, NFI = 0.94, NNFI = 0.96, GFI = 0.91, AGFI = 0.89, CFI = 0.96). The authors also sought its internal consistency and reported Cronbach’s alpha to be 0.89 and split-half reliability coefficients to be 0.89 (Spearman-Brown) and 0.82 (Guttman). It should be noted that a measurement with the RHAS necessitates reverse scoring for items 4, 6, 9, 10, 14, 16, 17, 18, and 19 (Özdil and Demir, 2020). We found the internal consistency reliability of the scale to be *α* = 0.85.

#### Optimism–pessimism questionnaire

Based on their meticulous examination of the relevant literature (Alansari and Kazem, 2008; Çalışkan and Uzunkol, 2018; Furlong et al., 2017; Scheier and Carver, 1985), Arslan and Yıldırım (2021) developed the 12-item Optimism-Pessimism Questionnaire (OPQ). The OPQ items are rated on a 5-point Likert-type scale ranging between 1 (strongly disagree) to 5 (strongly agree). In the pilot study with 138 undergraduate students, the authors confirmed the factorial structure of the scale with satisfactory data-model fit statistics (*χ^2^* = 228.52, df = 53, *p* < 0.001, CFI = 0.95, TLI = 0.94, RMSEA [95% CI] = 0.079 [0.069, 0.090]). The standardized factor loadings ranged from good to excellent (Optimism *λ* = 0.75–0.79 and Pessimism λ = 0.56–0.82). The internal reliability coefficient (*α*) was found to be 0.86 and 0.88 for the subscales, respectively. Subsequently, the authors designed the short version of the OPQ (OPQ–6) with three items with the highest factor loadings for each subscale. The confirmatory factor analysis was replicated for this short version, and the findings provided satisfactory data-model fit statistics (*χ^2^* = 19.30, df = 8, *p* = 0.013, CFI = 0.99, TLI = 0.98, RMSEA [95% CI] = 0.052 [0.022, 0.081]) with regression weights ranging between 0.77 and 0.84. Similar to the long version, the OPQ–6 also demonstrated strong internal reliability (Optimism *α* = 0.83 and Pessimism *α* = 84). In this study, we employed the Optimism subscale of the OPQ-6 and calculated its internal consistency to be *α* = 0.76.

#### Meaningful living scale

Developed by Arslan (2020), the Meaningful Living Scale (MLS) consists of six items. In the original study, the scale yielded a single-factor structure, accounting for 46% of the total variance with factor loadings between 0.58 and 0.82. In addition, CFA revealed that the measurement model of the MLS produced excellent data–model fit values. The scale also provided strong internal consistency, and we calculated Cronbach’s alpha for the scale to be *α* = 0.86.

### Statistical analysis

We carried out this study to explore the mediating role of optimism in the relation between reading habits and meaningful living among prospective Turkish language teaching students. The data are presented as number (n), percentage (%), mean (*M*), and standard deviation (*SD*). Prior to the analyses, we checked outliers, missing data, univariate (skewness-kurtosis values) and multivariate normality, and other assumptions of the respective tests (Çokluk et al., 2021). We used independent samples *t*-test and one-way analysis of variance (ANOVA) with Bonferroni correction to explore if participants’ meaningful living scores significantly differ by their gender and year of study, respectively. The relations between the research variables were first explored using Pearson’s correlation analysis. Then, the variables were recruited for multiple linear regression and mediation analyses to test our hypothesis. We performed all analyses on SPSS 26.0 and SPSS AMOS 24.0 and accepted a *p* < 0.05 statistically significant.

## Results

We found that the majority of participants (58.4%) were females, slightly more than half (52.0%) were aged 21–30 years, and 34% of them were second-year students. The demographic characteristics of participants are summarized in Table 1.

**Table 1:** Participants’ demographics.

Characteristic	*n*	%
Gender	Female	58.4
	Male	41.6
Age (year)	17–21	46.0
	21–30	52.0
	31–40	2.0
Year of study	1	18.8
	2	34.2
	3	19.8
	4	27.2

Our findings showed that female students (*M* = 34.21) exhibited significantly higher meaningful living compared to their male counterparts (*M* = 29.41; *t* = 4.386, *p* < 0.001). Nevertheless, the ANOVA results demonstrated no significant difference between participants’ meaningful living scores by their year of study [*F*_(3, 198)_ = 0.500, *p* = 0.683]. The results are outlined in Table 2.

**Table 2:** Participants’ meaningful living scores by gender and year of study.

Variable	Characteristic		*n*	*M*	*SD*	*t/F*	*p*
Meaningful living	Gender	Female	118	34.21	5.07	4.836	<0.001
		Male	84	29.41	8.01		
	Year of study	First year	38	33.18	6.80	0.500	0.683
		Second year	69	32.30	6.92		
		Third year	40	32.25	6.86		
		Fourth year	55	31.41	6.83		

Collecting research data using a single survey form encompassing self-report instruments may lead to common method bias. To minimize this effect, we initially anonymized the data collection tools and presented them to participants in different orders, which also helped reduce the order effect (Podsakoff et al., 2003). Then, we performed an exploratory factor analysis (EFA) on all items covered by the instruments to investigate the potential influence of common method bias. Accordingly, we discovered 6 factors with eigenvalues exceeding 1. The initial eigenvalue for the first factor was 7.269, accounting for 25.960% of the variance. As this value fell below the critical threshold of 40%, we may assert that our findings would be free of common method bias (Podsakoff et al., 2003). Table 3 presents descriptives of participant scores, univariate normality findings, and the results of the correlation analysis. As the data exhibited a normal distribution (skewness and kurtosis values ≤ ±1; Çokluk et al., 2021), we performed Pearson’s correlation analysis and found a weak significant relation between reading habits and optimism (*r* = 0.191, *p* = 0.007) and meaningful living (*r* = 0.269, *p* < 0.001). The findings also revealed a moderate, significant correlation between optimism and meaningful living (*r* = 0.628, *p* < 0.001).

**Table 3:** Descriptive statistics and findings of the correlation analysis.

Variables	*n*	Min.	Max.	*M*	*SD*	Skewness	Kurtosis	1	*p*	2	*p*	3	*p*
1. Reading habits	202	39.00	95.00	68.11	11.20	−0.122	−0.305	1	-	0.191	0.007	0.269	< 0.001
2. Optimism	202	3.00	15.00	9.63	2.67	−0.192	−0.300			1	-	0.628	< 0.001
3. Meaningful living	202	8.00	42.00	32.21	6.86	−0.951	0.546					1	-

Before regression analysis, we checked the relevant assumptions of the analysis. First, Multicollinearity refers to a high degree of correlation between two or more predictor variables in a regression model. It can inflate standard errors, making it difficult to determine the individual effects of each predictor and potentially leading to unstable or unreliable results (Tabachnick and Fidell, 2013). Although tolerance values for both predictors (0.964) imply some concerns about multicollinearity, the magnitudes of the associations between the variables (*r* < 0.90), the VIF values (1.038) and condition indices (<15) for the predictors suggest the absence of multicollinearity. Moreover, we ensured multivariate normality through the relevant graphs. The absence of homoscedasticity and the independence of residuals were checked through the scatterplot and the Durbin-Watson statistic (2.09), respectively (Field, 2009; Tabachnick and Fidell, 2013).

**Figure 1:**
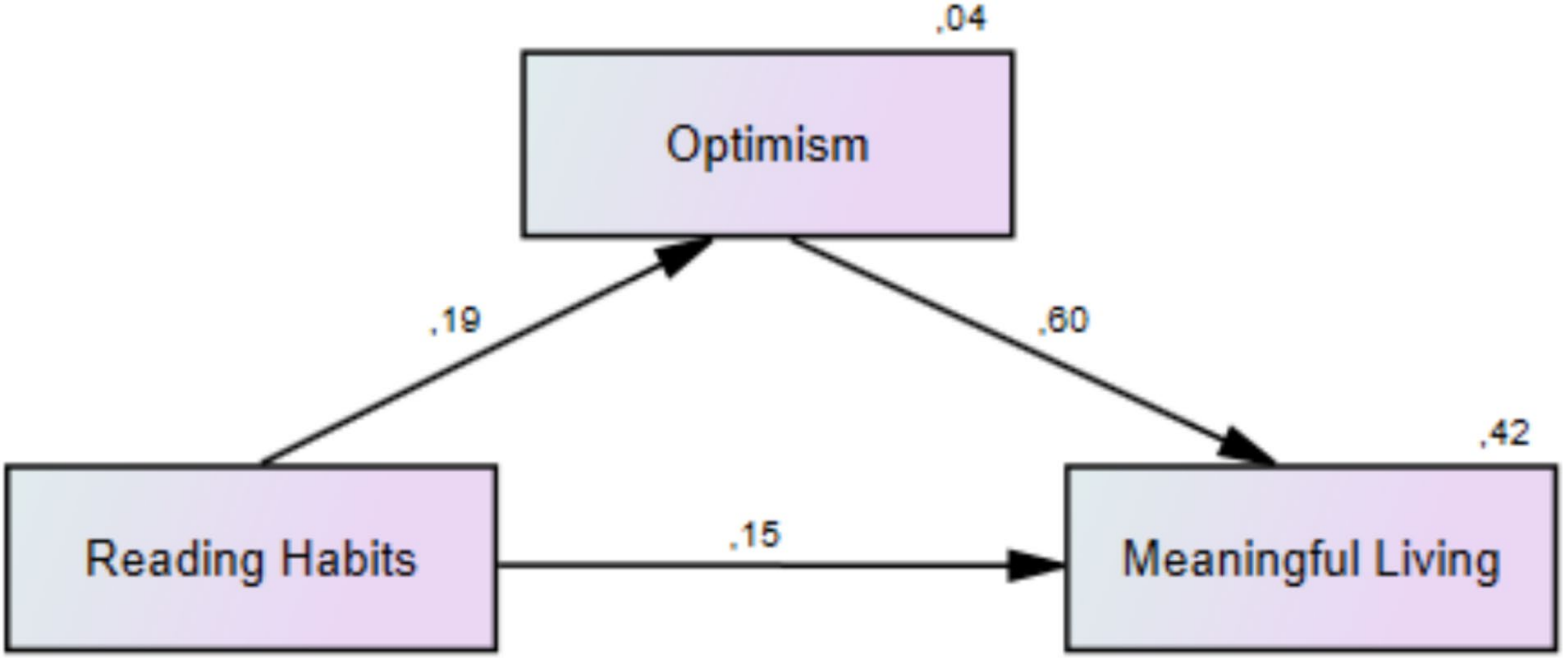
Conceptual framework of the mediating role of optimism in the relation between reading habits and meaningful living.

The analysis showed that the model built with the research variables was significant, and two predictor variables, reading habits and optimism, explained about 42% of the variance in optimism with a large effect size [*F*_(2, 199)_ = 71.413, *p* < 0.001; *R^2^* = 0.418; Cohen’s *f^2^* = 0.71]. The follow-up mediation analysis revealed a significant direct effect of reading habits on meaningful living (*β* = 0.154, 95% CI: 0.032, 0.154) and explained about 7% of the variance in meaningful living with a small effect size (Cohen’s *f^2^* = 0.07). In addition the model yielded the following values for baseline goodness-of-fit-indices: *χ^2^*/df = 20.54, *p* < 0.001, Root Mean Square Error of Approximation (RMSEA) = 0.06, Normed Fit Index (NFI) = 0.93, Relative Fit Index (RFI) = 0.90, Incremental Fit Index = 0.90, Tucker-Lewis Index (TLI) = 0.91, and Comparative Fit Index (CFI) = 0.90. Thus, it can be asserted that participants’ reading habits are linked to their levels of meaningful living. The indirect effect of reading habits on meaningful living through optimism was also significant (*β* = 0.114, 95% CI: 0.027, 0.108), suggesting that participants’ optimism is likely to assume a mediating role in the relation between their reading habits and meaningful living (Table 4). The effect of optimism can be contextually important: Even if the effect size of optimism is statistically small, this does not mean that optimism is unimportant in practical life. In particular, even a small effect of optimism can have important consequences in the context in which the study is conducted (e.g., individuals in difficult situations, those with chronic diseases, etc.). Figure 1 illustrates a graphical representation of the mediation model with the research variables.

**Table 4:** Mediating role of optimism in the relation between reading habits and meaningful living.

Type	Effect	Estimate	SE	95% CI (a)		*β*	*p*
				Lower	Upper		
Indirect	Reading habits ⇒ Optimism ⇒ Meaningful living	0.070	0.024	0.027	0.108	0.114	0.010*
Component	Reading habits ⇒ Optimism	0.046	0.015	0.018	0.191	0.191	0.006*
	Optimism ⇒ Meaningful living	1.537	0.143	1.289	0.599	0.599	< 0.001*
Direct	Reading habits ⇒ Meaningful living	0.095	0.034	0.032	0.154	0.154	0.005*
Total	Reading habits ⇒ Meaningful living	0.165	0.043	0.083	0.237	0.269	0.010*

Confidence intervals computed with the method: Bootstrap percentiles. Betas indicate standardized effect sizes.

## Discussion

This study advances the existing literature by integrating the role of optimism into the relationship between reading habits and meaningful living, an area previously underexplored. Unlike prior studies that primarily examined reading habits and wellbeing separately, this research establishes a direct link between reading and meaningful living through optimism. Additionally, the findings provide a culturally relevant perspective by focusing on Turkish university students, contributing to cross-cultural understandings of reading and wellbeing. Findings demonstrated positive associations between these variables and perceived meaningful living. Moreover, the indirect effect of reading habits on meaningful living through optimism may be considered a notable finding that contributes significantly to the existing knowledge in the literature. In this study, participants’ reading habits had a direct positive effect on their perceived meaningful living. The analysis results indicate that optimism partially mediates the relationship between reading habits and meaningful living. However, reading habits also directly influence the perception of a meaningful life. This finding suggests that reading not only enhances individuals’ levels of optimism but also directly helps them discover the meaning of life. In particular, reading processes may play a crucial role in developing critical thinking skills, increasing self-efficacy, and enabling individuals to evaluate life events more consciously. Future studies could explore this relationship in greater detail by considering additional mediating variables, such as psychological resilience and self-efficacy.

Similarly, previous research documented that reading habits facilitate adopting a broader perspective on life, fostering empathy, and enhancing self-understanding (Billington et al., 2017). Some previous studies have shown that optimism fully mediates the relationship between reading habits and meaningful living (Carver and Scheier, 2005). However, our study found only partial mediation. This difference may be attributed to cultural factors. In Turkey, reading habits may be limited to academic obligations, and individuals may rely on different social and cultural mechanisms in their search for meaning. Additionally, it should be considered that optimism levels in Turkey may be influenced by factors such as family structure, societal values, and religious beliefs. Future studies should examine the role of cultural factors in greater detail. Moreover, it was previously asserted that reading habits exert a modest yet substantial influence on the augmentation of perceived meaningfulness in life (Clark and Rumbold, 2006; Guthrie and Wigfield, 2000). Then, our findings underscore the notion that individuals with regular reading habits are likely to be better at formulating a more profound sense of meaning in their lives. Participants’ optimism was also positively linked with their perceived meaningful living. Overlapping with this finding, previous research showed that optimistic individuals may perceive challenging, undesirable life events as more manageable, which potentially leads them to pursue a more fulfilling life (Carver and Scheier, 2005). Besides, we found that optimism had a significant mediating role in the relation between reading habits and perceived meaningful living, which suggests that reading habits are likely to influence one’s adopting a positive perception of life through enhanced optimism. Similarly, the practice of reading is often considered to facilitate personal growth, offering one the opportunity to expand their worldview and enhance their psychological wellbeing. In this regard, reading habits were shown to contribute to the acquisition of knowledge while concurrently fostering a more positive outlook (Krashen, 2004).

Frankl’s (2021) logotherapy suggests that individuals seek meaning through both external factors (reading habits) and internal factors (optimism). In this context, our study’s findings indicate that individuals can strengthen their sense of meaning through both behavioral (reading) and cognitive (optimism) processes. Similarly, Seligman’s (2002) positive psychology approach emphasizes that optimism is central to psychological wellbeing. The partial mediation effect of optimism found in our study highlights that an individual’s perception of a meaningful life is influenced by both external and internal factors. Future research should further explore the intersection of logotherapy and positive psychology to better understand how individuals’ search for meaning can be supported.

Our findings showed that female participants exhibited higher levels of perceived meaningful living compared to their male counterparts, which aligns with previous research (Steger et al., 2006). Women’s higher meaningful living scores may be linked to social support structures and cultural expectations around emotional expression (Steger et al., 2006). Further research should investigate gender-specific influences on meaningful living. One potential explanation for this discrepancy may be attributed to higher levels of life satisfaction and psychological wellbeing among our female participants. Further research should investigate gender-specific influences on meaningful living.

Since both reading habits and optimism play a significant role in fostering a meaningful life, interventions designed to cultivate these traits may improve students’ overall psychological wellbeing. Universities should consider incorporating structured reading programs and workshops aimed at building optimism into their curricula. Furthermore, support systems like mentorship programs and peer discussion groups could further strengthen students’ capacity to find meaning in life. Future studies should investigate whether different types of reading materials (e.g., fiction vs. non-fiction) have distinct effects on optimism and meaningful living. Additionally, examining the long-term impact of sustained reading habits and optimism training through longitudinal studies would provide deeper insights. Exploring other moderating factors, such as personality traits and intrinsic motivation for reading, could further enhance our understanding of these relationships.

## Conclusion

The study provides empirical support for the role of reading habits and optimism in fostering meaningful living among university students. Enhanced reading habits have the potential to foster students’ self-understanding and worldview, while educational strategies that promote optimism can fortify their psychological wellbeing. From this perspective, it can be anticipated that educational programs tailored with a focus on reading-optimism and will contribute to students adopting a more positive perception of meaningful living.

## Implications, limitations, and recommendations

### Implications

The ultimate significance of the present study lies in its thorough examination of how reading habits and optimism interact in the development of perceived meaningful living among undergraduate students. Our findings underscore the dual contributions of reading habits to students’ cognitive development and their ability to pursue a meaningful life and fortify psychological wellbeing. In other words, reading habits not only facilitate cognitive development but also contribute to the emotional and social enrichment of life. In this study, we were also able to uncover the indirect role of reading habits in enhancing perceived meaningful living through optimism. Overall, it is prudent to assert that reading habits are associated with enhanced psychological wellbeing and satisfaction, enabling one to develop a more profound self-understanding and improve their ability to grasp their environment. Moreover, optimism is likely to facilitate one’s ability to enhance their wellbeing by cultivating their capacity to cope effectively with adverse life events.

Given the findings of this study, encouraging undergraduate students to engage in reading activities is likely to facilitate their self-understanding. Concurrently, cultivating optimism in students may promote their adoption of a more favorable outlook on life. Therefore, educators and psychologists should consider designing programs that promote reading and optimism, with the objective of enhancing students’ psychological wellbeing. In addition, the following suggestions can be made for educators and psychologists:

*Educational strategies*: universities should integrate structured reading programs into curricula to enhance students’ self-development and psychological resilience.

*Counseling applications*: psychological support services should incorporate reading interventions to foster optimism and a sense of purpose among students.

*Personal development programs*: institutions should develop workshops that encourage reflective reading practices, helping students connect literature with their life experiences and personal goals.

In a nutshell, our findings will pave the way for developing novel strategies to enhance psychological wellbeing and personal growth, as we were able to show substantial effects of undergraduate students’ reading habits and optimism on their perceived meaningful living. It should also be noted that this research and similar scholarly work may serve as the foundational basis for the development of life-enhancing psychological support services for those seeking meaning in their lives.

### Limitations

The principal limitation of this study may be that the findings are limited to the measurements from a sample of prospective Turkish language students. Further research may employ a sample covering diverse age groups and cultural contexts. In addition, prospective researchers may consider examining the impact of sub-dimensions of reading habits (e.g., reading specific literary genres) on perceived meaningful living. In this study, data were collected based on participants’ self-reports. This may lead to social desirability bias, as participants might consciously or unconsciously alter their responses to present themselves in a more favorable light. Future research could minimize such biases by employing objective measurement methods, such as digital tracking systems that assess reading habits or observation-based analyses. Additionally, longitudinal studies could be conducted to more robustly examine causal relationships between variables.

### Recommendations

*Encouraging reading habits*: our findings demonstrated that individuals engaging in regular reading habits are likely to have a heightened perceived meaningful living, underscoring the significance of reading habits not only for reinforcing academic achievement but also for personal growth and the pursuit of meaning in life. Therefore, there is a compelling rationale for the incorporation of reading habits into the curriculum in education faculties. Moreover, the promotion of reading activities and awareness-raising initiatives for diverse literary works can be instrumental in fostering a culture of reading. Integrating literary reading activities into university psychological counseling programs may help enhance students’ optimism levels and foster their perception of a meaningful life. For example, structured book reading groups could be established in universities and facilitated by psychological counselors. Additionally, to promote reading habits, book clubs and bibliotherapy programs could be designed around specific themes. Universities could also create “search for meaning” sections in libraries to increase students’ awareness of meaningful living.

*Promoting optimism*: the analyses showed optimism as a significant psychological factor that fortifies perceived meaningful living among undergraduate students. Therefore, positive thinking skills should be promoted in academic units and relevant counseling activities oriented to higher education students. In addition, supporting students’ self-confidence and ability to cope with undesirable situations (e.g., stress) is likely to contribute to their psychological wellbeing.

*Raising awareness of purpose and values in life*: as perceived meaningful living is linked with seeking a purpose and value in life, undergraduate students could be offered guidance and psychological counseling services to assist them in discovering unique purposes and values in their own lives.

*Increasing psychological counseling and guidance services*: we could reveal that optimism and reading habits positively influenced perceived meaningful living. Therefore, professionals may consider incorporating these topics in psychological counseling services to enhance undergraduate students’ psychological wellbeing. In addition, improving the visibility and accessibility of these services in campus settings will undoubtedly contribute to students’ psychological wellbeing.

*Advancing research with diverse demographic groups*: the findings of this study are limited to the measurements from a sample of prospective Turkish teachers. Research with students with different majors or diverse professional groups may provide more generalizable implications and broader social perspectives.

*In-depth examination of the relation between reading habits and optimism*: further research may scrutinize the interplay between reading habits and optimism to bring more profound explanations of how these variables affect one’s perceived meaningful living. Particularly, qualitative research may offer more insights into how reading experiences transform one’s perspectives on the world, thereby elucidating the profound effects of reading habits on psychological processes.

## Data Availability

The original contributions presented in the study are included in the article/supplementary material, further inquiries can be directed to the corresponding author.
